# Remimazolam for procedural sedation in the emergency department: a prospective study of effectiveness and patient satisfaction

**DOI:** 10.1186/s13049-025-01402-6

**Published:** 2025-05-20

**Authors:** Sofus Andreassen, Vibe Maria Laden Nielsen, Anne Lund Krarup, Annika Kamp, Dennis Møller Andersen, Steven Krogh-Larsen, Dorte Melgaard

**Affiliations:** 1 Department of Emergency Medicine and Trauma Centre, Aalborg University Hospital, Hobrovej 18-22, EMRUn, Aalborg, 9000 Denmark; 2 Department of Clinical Medicine, Aalborg University, Aalborg, Denmark

**Keywords:** Deep sedation, Benzodiazepines, Anaesthesia, Analgesia: emergency service, Hospital, emergency medicine

## Abstract

**Background:**

Remimazolam (RM) is a novel ultra-short acting benzodiazepine. This study evaluates the safety of using RM for procedural sedation in the emergency department (ED) comparing its administration by registered nurse anaesthetists versus house officers in 1st year residency in emergency medicine and emergency medicine physicians without previous anaesthesiologic specialisation. Secondary aims were patient satisfaction and proportion of successful procedures.

**Methods:**

This prospective study was performed at the ED at Aalborg University Hospital from 10 May through 20 August 2024. Five emergency medicine physicians (group 1) started administering RM to patients after completion of training and direct supervision. Results were compared to patients sedated by two registered nurse anaesthetists (group 2) who had been administering RM more than 50 times before study start. Time was recorded during sedation and a questionnaire filled out immediately after the patient had awakened. T-tests or Mann-Whitney U tests were used to compare groups. Proportions were calculated with chi-square (χ^2^) tests of proportion.

**Results:**

In group 1, 53 patients were sedated by emergency medicine physicians, and in group 2, 50 patients were sedated by registered nurse anaesthetists. No or mild respiratory adverse effects were observed in 97% of patients in group 1 versus 100% in group 2. Procedural amnesia was 93% in group 1 versus 90% in group 2. Patients were safe to be left unsupervised after a median of 15 min in both groups. Procedural success was 92% in group 1 versus 100% in group 2.

**Conclusions:**

Severe respiratory adverse effects after sedation were rare in both groups. Most patients had amnesia and adequate pain relief for the procedure. The use of RM by physicians without anaesthesiologic specialisation is considered a safe and effective alternative for procedural sedation in the ED.

**Trial registration:**

The study was registered and approved as a quality study (ID 2017–011259) by the hospital administration.

## Background

Procedural sedation for therapeutic and diagnostic purposes has had an increase in demand in the Emergency Department (ED) [1]. Procedural sedation aims to induce a state, where the patient can tolerate unpleasant procedures, as well as maintaining adequate cardiorespiratory function and protective airway reflexes [2]. Such sedatives and analgesics are mostly used by physicians and nurses with anaesthesiologic specialisation but are also in use by physicians without previous anaesthesiologic specialisation performing brief and moderate sedation in the ED [1] and in ambulatory settings [3–6]. Commonly used sedatives in such settings are Midazolam, Propofol, Ketamine, Dexmedetomidine and Remifentanil, each with their pros and cons [7]. Remimazolam (RM) is a novel ultra-short acting benzodiazepine. It has an onset of action of 1–3 min and has a plasma half-life of 10 min. It is an ester-based benzodiazepine and is rapidly hydrolysed into inactive metabolites. These metabolic qualities give a moderate sedation for a short duration of time and rapid recovery [8, 9]. Recent studies comparing RM with Propofol and Midazolam outside of the operating room show no difference in sedation efficacy, but faster recovery and shorter stay in the hospital for patients receiving RM [7–9]. Studies investigating safety find RM to be as safe as Midazolam and Propofol when applied for procedural sedation and general anaesthesia [10]. Similar to other benzodiazepines, potential side-effects of RM are hypotension, hypoventilation, bradycardia, and risk of aspiration.

This prospective observational clinical cohort study aimed to investigate the safety of using RM for sedation in the ED, comparing its administration by health professionals with or without specialisation in anaesthesia. Secondary aims included patient satisfaction and effectiveness of sedation and procedure.

## Methods

This clinical prospective cohort study was conducted from 10 May to 20 August 2024, at the ED at Aalborg University Hospital, North Denmark Region. Approximately 58,000 patients enter the ED annually (2023), including 23,000 orthopaedic surgery patients and 35,000 from other specialties. Around 550 trauma calls are handled annually.

House officers in 1st year residency in emergency and medicine Emergency medicine physicians without previous anaesthesiologic specialization, but well trained in handling airway management were qualified to administer RM after a three-step training program. (The two groups of doctors are hereafter referred to as emergency medicine physicians)

1. Emergency medicine physicians received a thorough oral and written introduction to RM administration, including pre-sedative and sedative procedure, pharmacodynamic properties of RM and handling of potential adverse effects from a registered nurse anaesthetist, and observed experienced nurse anaesthetists performing RM sedation.
2. Emergency medicine physicians administered RM under direct supervision of registered nurse anaesthetists and were qualified to administer RM unsupervised after three successful administrations.
3. Emergency medicine physicians performed sedation with RM unsupervised with experienced staff present.

### In- and exclusion criteria

Patients were included if they were (1) aged ≥ 12 years, (2) American Society of Anaesthesiologist physical status classification (ASA) score l-lll or IV with the approval of the on-call anaesthesiologist, and (3) in need of a procedure expected to cause pain or anxiety.

Exclusion criteria were ABCDE instability prior to procedure or had a known medication allergy for benzodiazepines, were influenced by alcohol or had myasthenia gravis.

## Outcome measures

The sedation was performed by emergency medicine physicians without previous anaesthesiologic specialisation (group 1) and compared with procedures performed by registered nurse anaesthetists with experience in RM sedation (group 2). Outcome measures were (1) safety; occurrence of respiratory problems graded as mild/moderate if the respiratory problem was corrected by turning up the oxygen flow, performing jaw lift or inserting a naso/pharyngeal airway. If the patient had bag-valve-mask ventilation performed or were given an antidote, the respiratory problem was defined as severe. (2) patient and provider satisfaction; proportion of patients having amnesia for the procedure, exhibiting no signs of pain during the procedure (waking up, moaning, facial expressions of pain) and the procedure sedation subjectively evaluated as a success by the provider. (3) sedation and procedural effectiveness; reported as time from first dosage administered to the procedure could be started and proportion of patients sleeping when the procedure started, were safe to leave alone within 20 min. Based on clinical experience, it was also noted whether we would previously have booked an operating room (OR) for general anaesthesia, such as in patients with hip dislocations, where it is rarely possible to sufficiently relax the patient with midazolam to reduce the hip.

## Data collection

Demographics and medical data included: age, gender, height, weight, body mass index (BMI), ASA score, and opioids given up to 60 min before sedation. Type of procedure (e.g., reduction of dislocated joint, fracture manipulation, pleuracentesis), administration of relevant drugs (RM, alfentanil, fentanyl and morphine) and dosage was registered. Dosages were reported in milligrams (mg) or micrograms (µg).

## Procedure

### Preprocedural assessments

According to local guidelines [11], we evaluated patients’ ASA score, BMI, neck mobility and inspected for presence of large tongue. History concerning sleep apnoea and prior difficult airway was investigated. We performed preoxygenation 3 L/min and used continuous monitoring of SpO_2_, blood pressure every fifth minute and continuous heart rhythm. Opioids were administered after initiation of sedation for fracture manipulations or correction of dislocated joints, as well as other procedures where intraprocedural pain was expected.

### Intraprocedural assessments

Initial dosage of RM was 2.5-5.0 mg, followed by refractory dosages of 2.5 mg RM until the patient was in a state sedation where the procedure could be performed. Initial dosage depended on patients age, weight, comorbidity and preprocedural pain [12]. In case of insufficient sedation and analgesia, additional dosages of 2.5 mg RM and/or alfentanil 0.25-1.00 mg could be administered. The following equipment was readily available in case of complications: nasopharyngeal airway, suction catheter, ventilation bag and antidotes (Naloxone and Flumazenil) and atropine for bradycardia. In case of decreased blood oxygen saturation < 92%, the following standard actions should be done: Talking to the patient, gently shaking their shoulder, increasing oxygen flow, initiation of the planned painful procedure, jaw thrust, insertion of a naso- or oropharyngeal airway, administration of an antidote, or bag-valve-mask ventilation.

### Postprocedural assessments

Patients were monitored by the provider until fully awake and habitual blood oxygenation and respiratory frequency. Ten minutes after waking up, the patients were asked if they had any experience of pain or remembered the procedure, as well as their satisfaction level with the sedation and procedure.

### Statistical analyses

Descriptive statistics were reported as medians and inter quartile ranges. T-tests or Mann-Whitney U tests were used to compare groups as appropriate. Proportions were calculated out of all participating patients in each group unless otherwise stated and compared using the Chi-squared test of proportion. Data were collected and stored in REDCap, an electronic data capture software (REDCap, Version 9.5.6, Vanderbilt University) [13, 14]. SAS enterprise was used for data analyses (SAS Institute Inc., Cary, NC, USA). A p-value < 0.05 was considered statistically significant.

## Results

Descriptive characteristics of the 103 included patients are shown in Table 1. Five individuals emergency medicine physicians sedated 53 of the patients (52%), while the remaining 50 patients were sedated by two registered nurse anaesthetists. The most frequent procedures were joint reductions and fracture manipulations (Table 1).

**Table 1:**
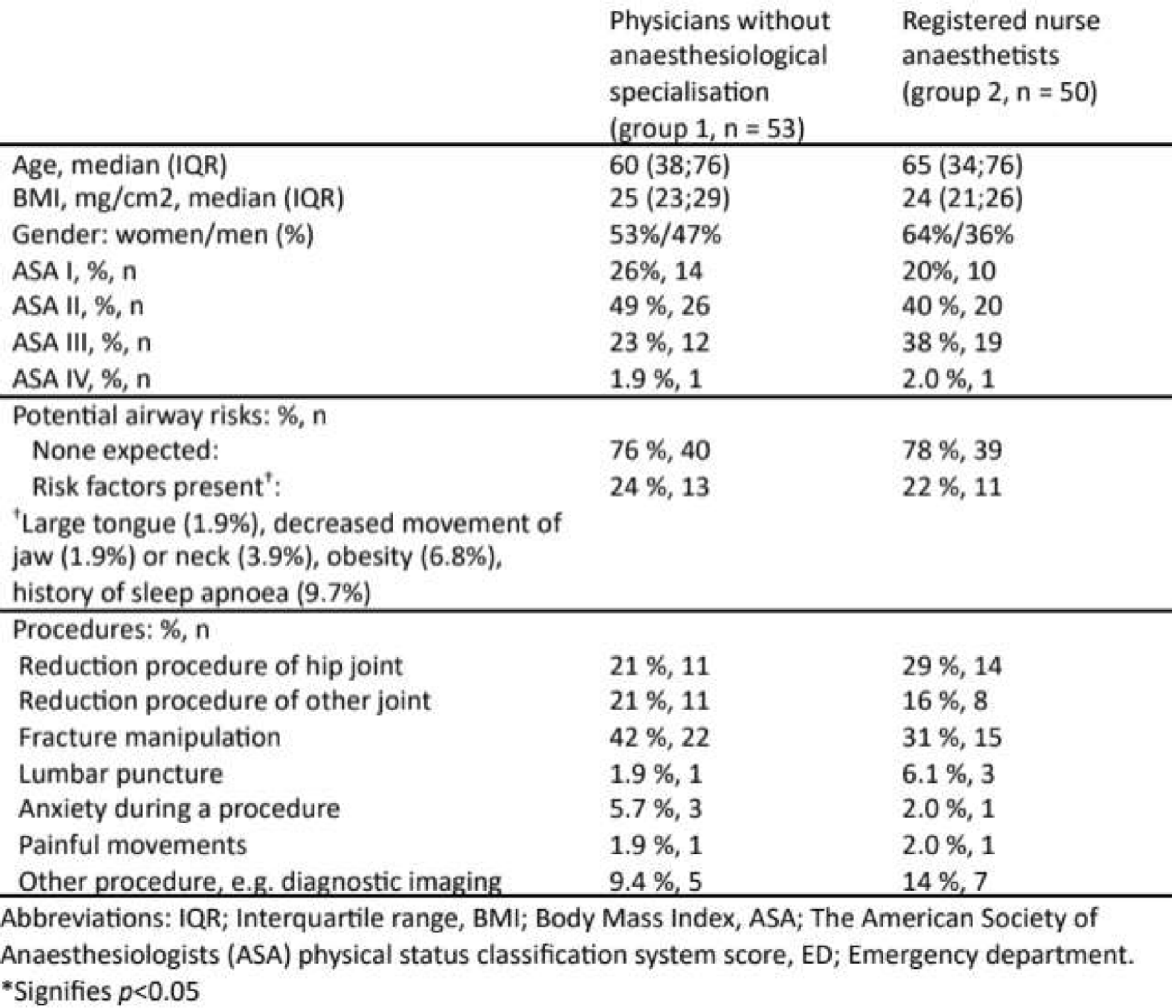
Patient characteristics by provider group, N= 103

### Remimazolam was safe to use, and severe respiratory adverse effects were rare

**Fig. 1:**
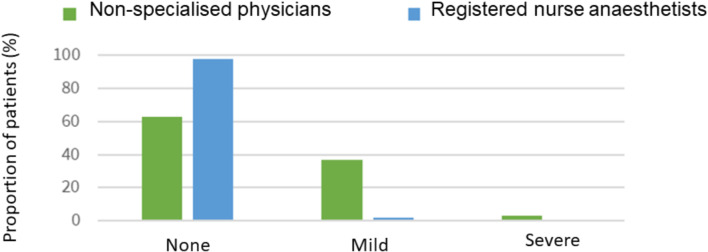
Safety. Respirator, problems dunng remimarrearn sedation performed bedsitters without specialisation in aneestiwsblogy (green) and nurse specialised in anaesthesiology thiue)

Total dosage interval of RM was 2.5–53.5 mg (Table 2). Sedation was performed without any respiratory adverse effects in most patients across both provider groups (total 78%) (Fig. 1).

**Table 2:**
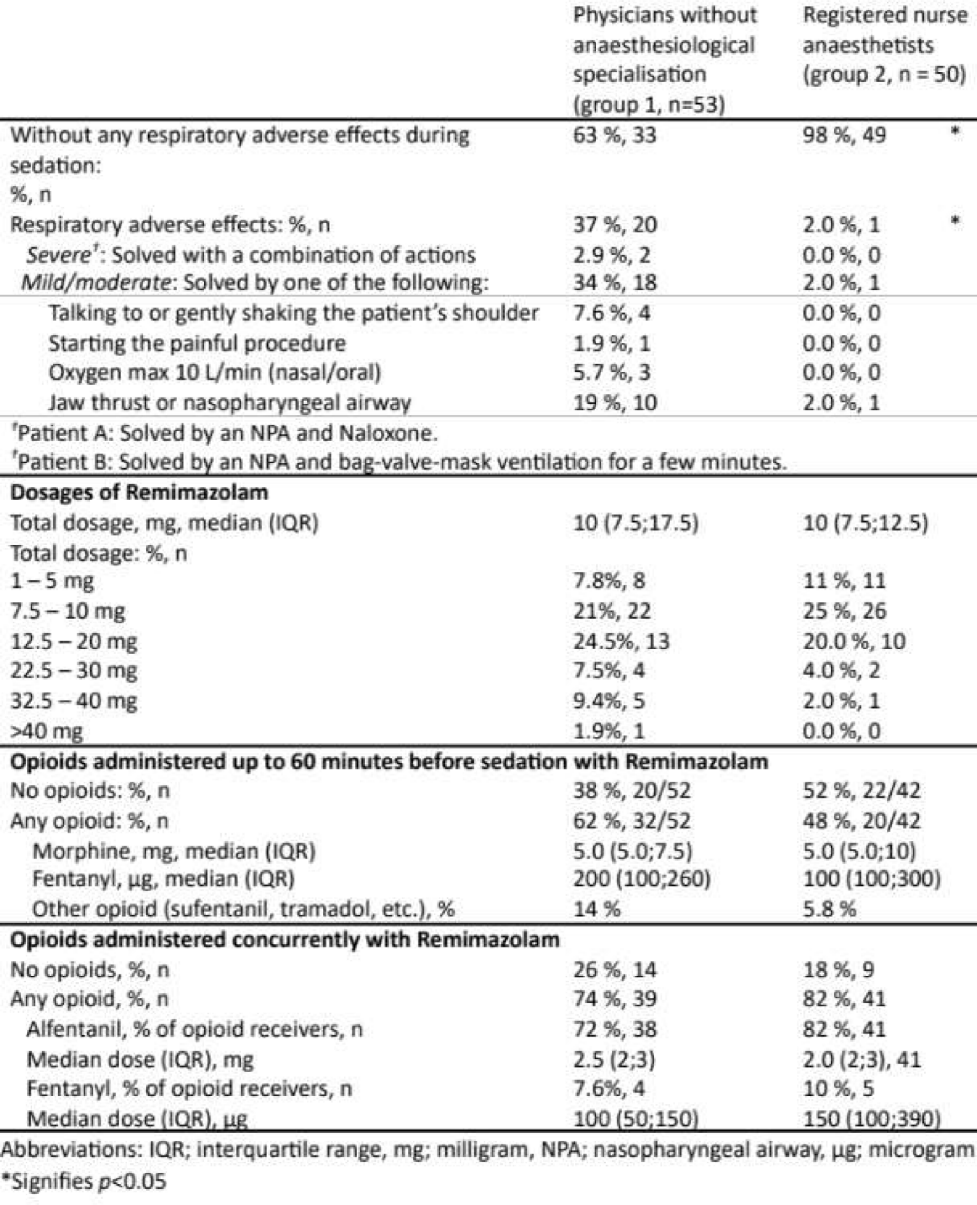
Adverse effects and required dosages of Remimazolam for procedural sedation in the ED by provider group, N= 103

In the 20% (*n* = 21) of patients where respiratory challenges did occur, all but two were short-lasting and mild to moderate in character (Table 2). Both patients with respiratory adverse effects were scored as ASA II, neither had had any respiratory symptoms prior to the sedation, and both had been sedated with a low dosage of RM (7.5–10 mg) by a physician without anaesthesiologic specialisation. For Patient A, no previous opioids had been given. The patient had a BMI > 30 but no history of snoring or sleep apnoea. 1 mg alfentanil was administered concurrent to the RM. After the joint had been relocated, the patient relaxed in the pharynx to a degree where a nasopharyngeal airway was necessary, and a single dose of Naloxone was administered. The patient woke up immediately.

Patient B had a normal BMI and had received 100 µg fentanyl in the prehospital setting. 1 mg alfentanil was administered right before RM was administered. After the dislocated fracture had been set, the patient relaxed to a degree where a nasopharyngeal airway and bag-valve-mask ventilation was necessary. The patient woke up a few minutes, before an antidote was administered.

### Patient and provider satisfaction was high

Most patients had amnesia for the procedure (96%), however among those who *did* remember the procedure, all were satisfied with the sedation (Table 3).

**Table 3:**
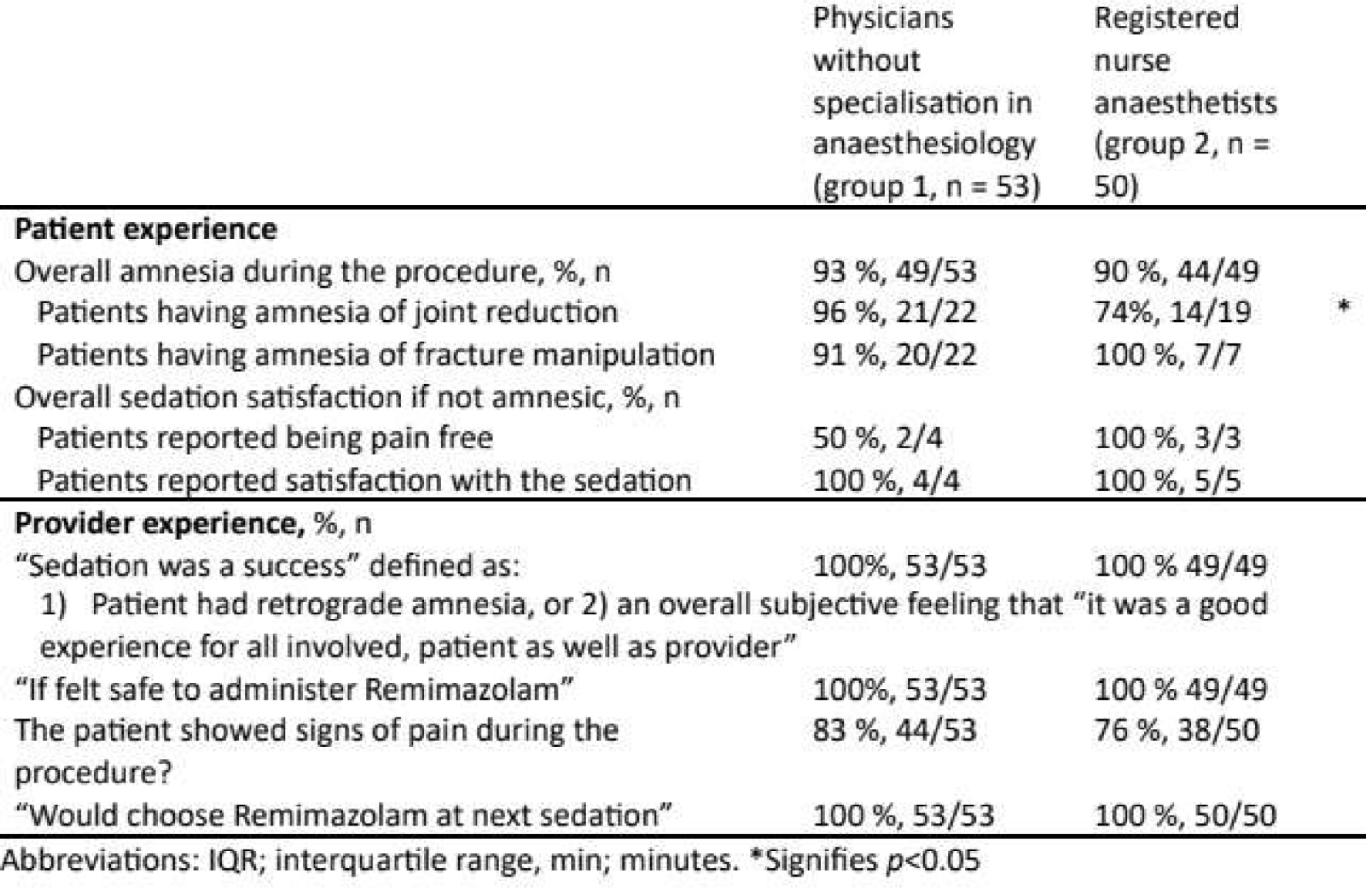
Patient and provider experiences by provider group, N= 103

**Fig. 2:**
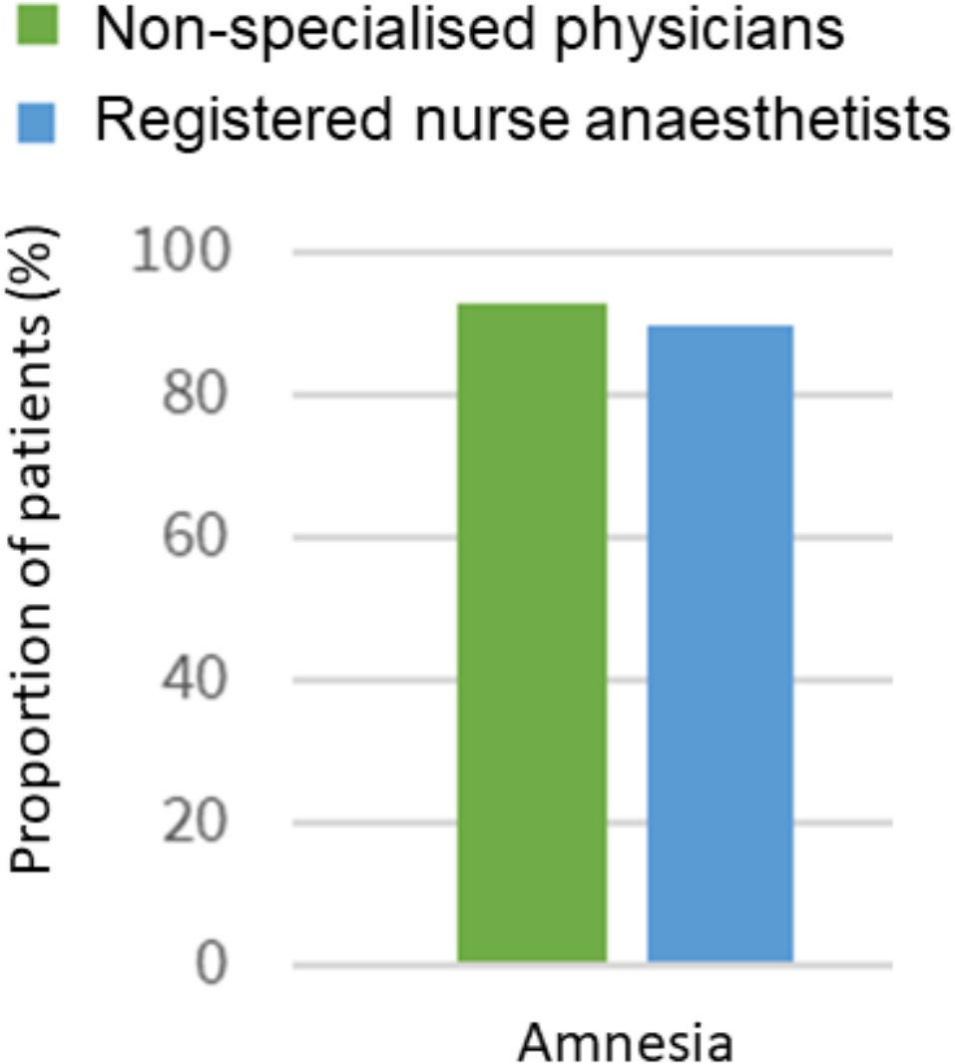
Patient satisfaction. Patient satisfaction during Renensarolam sedation performed by doctors without specialization in anaesthesiology (green/and nurses specialized In anaesthesiology (blue)

Overall, 100% of the procedures were subjectively evaluated by the provider as a “success for all of the involved”. The provider had observed an objective pain response in 41% of all patients, however, all but two patients reported that they did not remember having felt any pain during the procedure.

### Effectiveness of sedation and procedural success

**Fig. 3:**
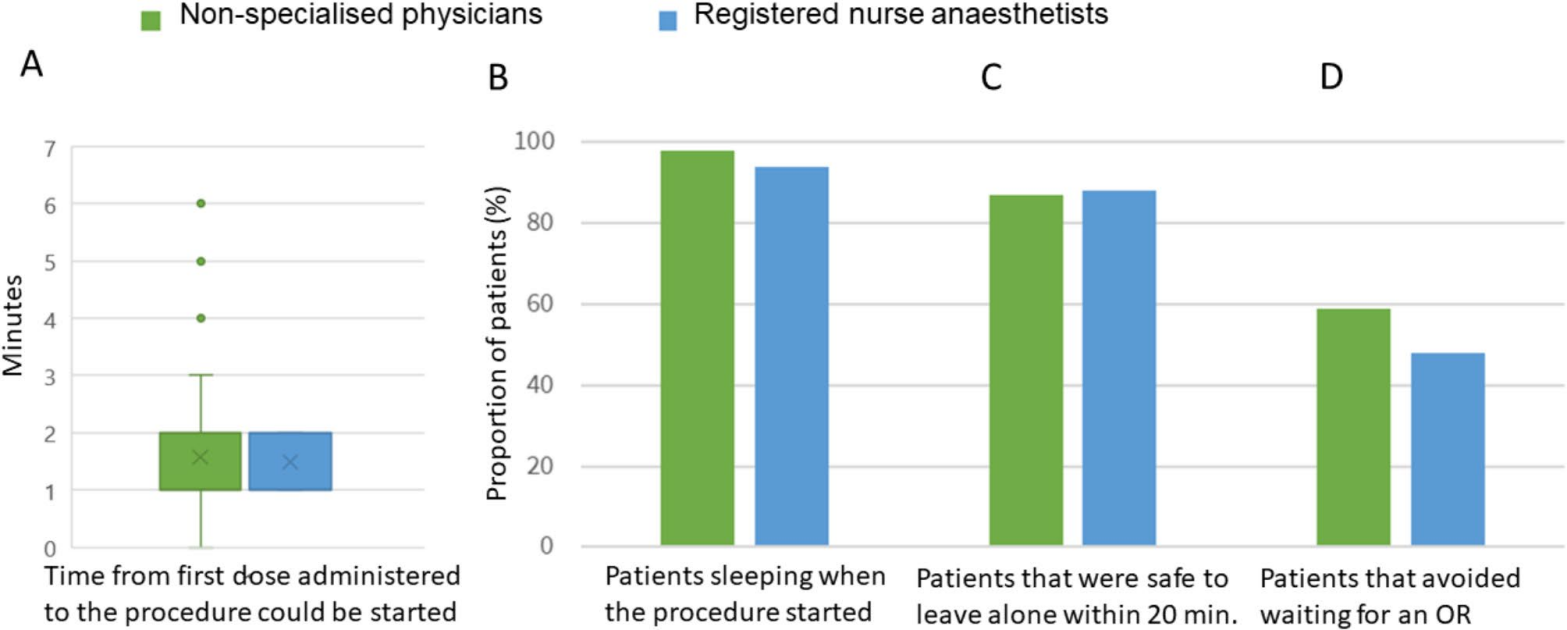
Sedation and effectiveness. Sedation and procedure effeNvencs, **A**) shows the time (minutes) from the first dose was administrated to the patient were sedate enough for the procedure to begin, **B** shows the proportion of patients that were sleeping when the paccdure started, **C**) that could safely be unmonitored within 20 minutes, **D**) and that we would previously have booked an operating men far, resulting in long waiting time In wed for the patient

Effective sedation occurred within a median of 1 min (IQR 1;2 min) in both groups, and the patient was awake and safe to leave unsupervised after a short period of time (Table 4; Fig. 3). Across both groups, 94% of procedures were successful (97/103), and 53% of patients that would previously have been transferred to the operating room for general anaesthesia, were instead successfully treated in the ED under sedation with RM.

**Table 4:**
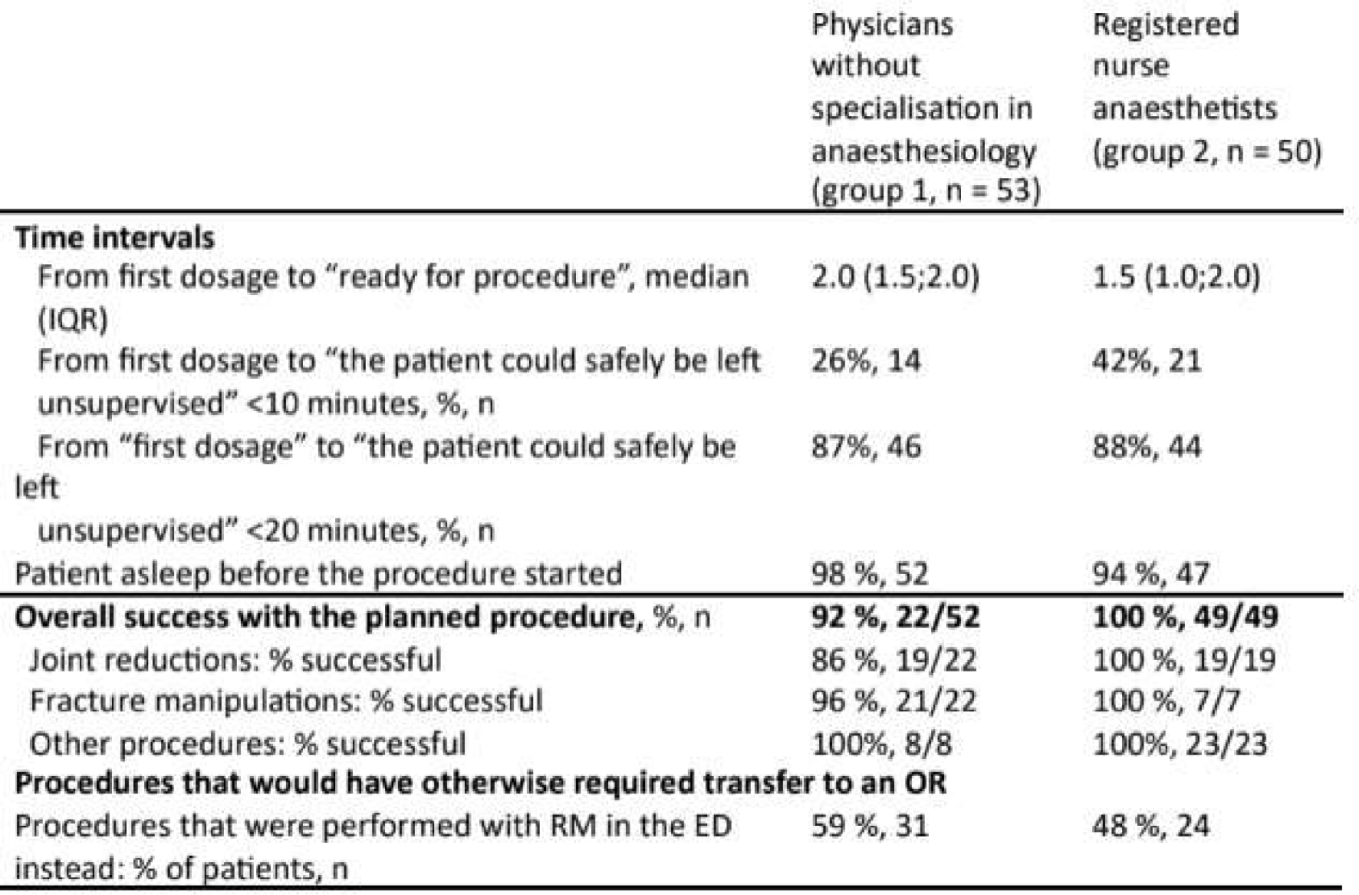
Effectiveness by provider group, N= 103

## Discussion

During procedural sedation with RM administered by emergency medicine physicians or nurse anaesthetists, two out of 103 patients experienced severe respiratory adverse effects that could be effectively treated in the emergency department. Patients were safe to leave unsupervised after a median of 15 min in both groups, and procedural success was high in both groups.

In this pragmatic clinical study, the incidence of any adverse events is reported. Many patients received opioids during the procedural sedation with RM (26% in group 1/emergency medicine physicians and 18% in group 2/nurse anaesthetists).

Only two patients developed severe respiratory adverse effects, and in both patients, symptoms could be resolved in the emergency department by simple airway manoeuvres, bag-valve-mask ventilation for a short period of time and/or administration of an antidote. These procedures are all well known by the staff in E.Ds. The proportion of patients requiring a manual airway manoeuvre is lower than in a recent prospective observational study of adult patients receiving RM in an ED in the Netherlands [15].

Amnesia was reported in most patients which is desired for patients undergoing painful procedures. While most patients showed objective signs of pain during the procedure, this had minimal impact on their overall satisfaction, as nearly all patients had amnesia for the procedure. These findings are consistent with those of Yao et al., who also reported a high patient satisfaction after sedation with RM during colonoscopy [16].

There were positive experiences with all attempted procedures. Several of the colleagues, who performed the painful procedures on the patients, expressed ability to more easily perform the procedures. They attributed this to their experience of improved sedation, better patient relaxation, and reduced time consumption due to easier adjustment of sedation and anesthetic depth. However, data has not been collected regarding this. Providers expressed confidence in the safety of administering RM [16, 17].

Time from first dosage until 87% of emergency medicine physicians without anaesthesiologic specialisation and 88% of registered nurse anaesthetists could safely leave patients unsupervised was less than 20 min. Our findings on the rapid onset and recovery times are consistent with an existing study on the sedative effect of RM [17]. The rapid recovery time and no active metabolites makes RM useful in the ED, as patients can be discharged earlier than after general anaesthesia.

Sancheti et al. states the following essential properties for sedatives: rapid onset, short half-life and pharmacologic reversibility, minimal impact on cardiac and respiratory function and lack of accumulation with sustained use as well as reliance on a particular organ metabolism [18]. The abovementioned properties were consistent with our findings concerning the safety and effectiveness of RM. The ED treats a diverse patient population with various comorbidities, enhancing the generalisability of our findings to other settings. However, in our opinion, it is essential that the setting has an on-call anaesthesiologist.

### Strengths and limitations

The study was conducted in a non-randomised clinical setting with consecutive patient inclusion, where the assignment to nurse or physician was based on provider availability during shifts. No formal power analysis was conducted, as this was an observational study based on the availability of patients receiving Remimazolam in our ED during the study period. All patients undergoing procedural sedation with RM during the study period were included, which minimised selection bias during the inclusion process. Providers were attending clinical duty during the inclusion and sedation of the patients. While this design offers certain advantages, it has notable limitations, particularly the lack of comparison to “standard care” scenarios, such as sedation in the emergency department using Alfentanil + Midazolam or nerve blocks and admission to an orthopaedic department while awaiting operating room availability Another limitation is that, due to the real-world nature of this study, a strictly matched-cohort analysis was not feasible.

A notable limitation is that patient satisfaction was assessed by the administering provider, potentially introducing treatment provider bias and a response bias. Additionally, the outcome “provider experience” is susceptible to outcome-assessor bias, as the same individual who performed the procedural sedation also evaluated their own experience.

## Conclusions

Severe respiratory adverse effects were rare in both groups. The vast majority of patients receiving Remimazolam experienced amnesia and adequate pain relief during the procedure. Most patients could be safely left unsupervised within 20 min post-procedure. Half of the procedures performed in the ED using Remimazolam would otherwise have required general anaesthesia in an operating room setting.

These findings demonstrate that Remimazolam is a safe and effective alternative for procedural sedation in the ED, administered by emergency medicine physicians or registered nurse anaesthetists. Remimazolam’s rapid onset, short recovery time, and predictable pharmacokinetics make it particularly advantageous in the fast-paced ED environment, where minimizing patient wait times and procedural delays is crucial. The ability of non-anaesthesiological emergency medicine physicians and registered nurse anaesthetists to safely administer RM further underscores its feasibility for broader clinical implementation.

## Data Availability

No datasets were generated or analysed during the current study.

## Abbreviations

ASA: American Society of Anaesthesiologists physical status classification system
BMI: Body mass index
ED: Emergency department
ICU: Intensive care unit

## References

[1] Chawla N, Boateng A, Deshpande R. Procedural sedation in the ICU and emergency department. Curr Opin Anaesthesiol. 2017;30:507–12. 10.1097/ACO.0000000000000487. 28562388 10.1097/ACO.0000000000000487

[2] Godwin SA, Burton JH, Gerardo CJ, et al. Clinical policy: procedural sedation and analgesia in the emergency department. Ann Emerg Med. 2014;63:247–e5818. 10.1016/j.annemergmed.2013.10.015. 24438649 10.1016/j.annemergmed.2013.10.015

[3] Pastis NJ, Yarmus LB, Schippers F, et al. Safety and efficacy of remimazolam compared with placebo and Midazolam for moderate sedation during bronchoscopy. Chest. 2019;155:137–46. 10.1016/j.chest.2018.09.015. 30292760 10.1016/j.chest.2018.09.015

[4] Rex DK, Bhandari R, Desta T, et al. A phase III study evaluating the efficacy and safety of remimazolam (CNS 7056) compared with placebo and Midazolam in patients undergoing colonoscopy. Gastrointest Endosc. 2018;88:427–e4376. 10.1016/j.gie.2018.04.2351. 29723512 10.1016/j.gie.2018.04.2351

[5] Matsuda Y, Karino M, Kanno T. Relationship between the functional oral intake scale (FOIS) and the Self-Efficacy scale among Cancer patients: A Cross-Sectional study. Healthc (Basel Switzerland). 2020;8. 10.3390/healthcare8030269. 10.3390/healthcare8030269 PMC7551334 32823778

[6] Shi W, Cheng Y, He H, et al. Efficacy and safety of the Remimazolam-Alfentanil combination for sedation during gastroscopy: A randomized, Double-blind, Single-center controlled trial. Clin Ther. 2022;44:1506–18. 10.1016/j.clinthera.2022.09.014. 36763995 10.1016/j.clinthera.2022.09.014

[7] Barends CRM, Absalom AR, Struys MMRF. Drug selection for ambulatory procedural sedation. Curr Opin Anaesthesiol. 2018;31:673–8. 10.1097/ACO.0000000000000652. 30124543 10.1097/ACO.0000000000000652

[8] Chang Y, Huang Y-T, Chi K-Y, et al. Remimazolam versus Propofol for procedural sedation: a meta-analysis of randomized controlled trials. PeerJ. 2023;11:e15495. 10.7717/peerj.15495. 37334113 10.7717/peerj.15495 PMC10269568

[9] Tang Y, Yang X, Yu Y, et al. Remimazolam versus traditional sedatives for procedural sedation: a systematic review and meta-analysis of efficacy and safety outcomes. Minerva Anestesiol. 2022;88:939–49. 10.23736/S0375-9393.22.16631-9. 35785930 10.23736/S0375-9393.22.16631-9

[10] Hu Q, Liu X, Wen C, et al. Remimazolam: an updated review of a new sedative and anaesthetic. Drug Des Devel Ther. 2022;16:3957–74. 10.2147/DDDT.S384155. 36411859 10.2147/DDDT.S384155 PMC9675580

[11] Møller JM. Sedation of patients using Remimazolam and Alfentanil in the emergency department. 2024.https://pri.rn.dk/document/AALBORGUH-905462050-26672 (accessed 6 Jan 2025).

[12] Ejstrud P, Aalborg UH. Sedation of patients with remimazolam without the involvement of anaesthetic personnel. 2023.https://pri.rn.dk/Sider/34629.aspx

[13] Harris PA, Taylor R, Thielke R, et al. Research electronic data capture (REDCap)-A metadata-driven methodology and workflow process for providing translational research informatics support. J Biomed Inf. 2009;42:377–81. 10.1016/j.jbi.2008.08.010. 10.1016/j.jbi.2008.08.010 PMC2700030 18929686

[14] Harris PA, Taylor R, Minor BL, et al. The REDCap consortium: Building an international community of software platform partners. J Biomed Inf. 2019;95:103208. 10.1016/j.jbi.2019.103208. 10.1016/j.jbi.2019.103208 PMC7254481 31078660

[15] van der Have A, ten Harmsen BL, van Storm BW, et al. Remimazolam for procedural sedation: a future sedative potential in the emergency department? Emerg Med J. 2024;41:586–7. 10.1136/emermed-2023-213805. 38811144 10.1136/emermed-2023-213805

[16] Yao Y, Guan J, Liu L, et al. Discharge readiness after remimazolam versus Propofol for colonoscopy: A randomised, double-blind trial. Eur J Anaesthesiol. 2022;39:911–7. 10.1097/EJA.0000000000001715. 35796575 10.1097/EJA.0000000000001715

[17] Rex DK, Bhandari R, Lorch DG, et al. Safety and efficacy of remimazolam in high risk colonoscopy: A randomized trial. Dig Liver Dis. 2021;53:94–101. 10.1016/j.dld.2020.10.039. 33243567 10.1016/j.dld.2020.10.039

[18] Sancheti S, Uppal V. Is remimazolam the future of sedation for regional anesthesia? Can J Anaesth. 2024;71:731–6. 10.1007/s12630-024-02697-2. 38378938 10.1007/s12630-024-02697-2

